# Research progress on the role of dendritic cells in glioma during 1992-2024: a bibliometric analysis

**DOI:** 10.3389/fimmu.2025.1510549

**Published:** 2025-06-20

**Authors:** Linglei Zhu, Linpeng Zhang, Shuqi Han, Xuan Zhou, Hai Pan, Fanghe Li, Kuo Gao

**Affiliations:** ^1^ Beijing University of Chinese Medicine, Beijing, China; ^2^ Department of Neurosurgery, Sanbo Brain Hospital, Capital Medical University, Beijing, China

**Keywords:** dendritic cells, glioma, bibliometric analysis, visualized, CiteSpace, VOSviewer

## Abstract

**Background:**

Gliomas represent the most prevalent primary neoplasms of the central nervous system. Activating an immune response by dendritic cells is pivotal in glioma immunotherapy. This study offers a comprehensive bibliometric analysis to elucidate the role of dendritic cells in gliomas.

**Method:**

We extracted literature related to glioma and dendritic cells from 1992 to 2024 using the Web of Science Core Collection. Utilizing CiteSpace, Vosviewer and Microsoft Excel, we analyzed the volume of publications, the contributing countries/regions, institutions, authors, journals, references and keywords.

**Results:**

A total of 1,576 articles were included, revealing an annual surge in dendritic cell-focused glioma research. The USA, China and Germany were the leading countries in publication output. Okada, Hideho had the most publications, while Stupp, R had the highest co-citations. Journal of Neuro-Oncology published the most articles, and Cancer Research received the highest citations. The analysis highlights pivotal themes including “dendritic cell”, “immunotherapy”, and “glioblastoma”, alongside emerging areas of interest such as “tumor microenvironment”, “immune infiltration” and “double blind”. Notably, the exploration of dendritic cell vaccinations is a key area of glioma therapeutic research, and there is growing interest in it.

**Conclusion:**

This study conducts a bibliometric analysis of publications related to dendric cells in glioma. Our findings suggest that dendritic cells, immunotherapy and glioblastoma will remain the focal points and emerging trends in dendritic cell-glioma research, providing valuable insights for future studies. Dendritic cell vaccines show promise in glioma trials but are hindered by the immunosuppressive tumor microenvironment. Future work should enhance dendritic cell function and explore combination therapies to improve outcomes.

## 1 Introduction

Gliomas, particularly glioblastoma (GBM), have been regarded as intractable malignancies due to their high invasiveness and propensity for recurrence. Although conventional therapies such as surgery, radiotherapy and chemotherapy have to some extent extended patient survival, their efficacy remains limited. For instance, the median survival with radiotherapy alone is only 12.1 months, and the recurrence rate is extremely high (1). In recent years, immunotherapy has emerged as a novel approach in cancer treatment and has gradually become a research focus in the management of gliomas due to its specificity and relatively lower side effects (2).The core principle of immunotherapy lies in activating or enhancing the patient’s own immune system to recognize and attack tumor cells. The main research directions in glioma immunotherapy currently include immune checkpoint inhibitors (such as PD-1/PD-L1 and CTLA-4 inhibitors), CAR-T cell therapy, tumor vaccines, and oncolytic viruses (3).The development of immunotherapy for gliomas has engendered new hope for the treatment of this refractory disease. However, further in-depth research is still needed in aspects such as target selection, technological optimization, and clinical trial design to realize its widespread application.

Dendritic cells (DCs) are pivotal antigen-presenting cells in the immune system, responsible for initiating and modulating adaptive immune responses. By capturing, processing, and presenting antigens, DCs activate T cells and B cells, thereby playing a crucial role in anti-infection, anti-tumor immunity, and the regulation of autoimmune responses (4). From a clinical perspective, DCs have also demonstrated significant potential in tumor immunotherapy. For instance, autologous dendritic cell vaccines have been shown to elicit immune responses and activate T cells targeting tumor antigens (5). In the treatment of glioma, a highly invasive central nervous system tumor with limited efficacy from traditional therapies (1), dendritic cell-based immunotherapy strategies offer new hope for patients.

Compared to other immune cells, such as macrophages or B cells, DCs possess superior capabilities in antigen presentation and immune activation (6). Given their central role and potent functions within the immune system, DCs have become the preferred cell type for the immunotherapy of glioma. In the tumor microenvironment (TME) of glioma, DCs play a dual role. Under healthy conditions, DCs, as antigen-presenting cells, can activate helper T cells, cytotoxic T cells, and natural killer (NK) cells (7). Thus, DCs exhibit anti-tumor activity by initiating immune responses against tumor antigens. However, within the glioma environment, the STAT3 signaling pathway inhibits the maturation of myeloid cells into DCs, leading to the formation of tumor-associated dendritic cells (TADCs). These immature DCs fail to sensitize the immune system to the growing tumor, thereby promoting immune suppression (8). In summary, DCs have emerged as a key target for the immunotherapy of glioblastoma. Despite the immunosuppressive factors in the tumor microenvironment that challenge their function, the antigen-presenting capacity of DCs can be enhanced by blocking inhibitory signals or activating key targets, thereby significantly improving the efficacy of immunotherapy. As research in this field continues to advance, there is optimism that the application of DC-based immunotherapy will yield more effective treatment options and better prognoses for patients with glioma.

Bibliometrics, the quantitative analysis of knowledge carriers using mathematical and statistical methods, provides valuable insights into the global development of a field by visualizing data from countries, institutions, journals, authors and more (9). Furthermore, it can evaluate and predict foundational and emerging research trends by the analysis of reference and keyword co-occurrence and emergence (10). VOSviewer and CiteSpace are two widely used bibliometric analysis software tools (11). This study employs visual analysis via VOSviewer and CiteSpace to elucidate research progress on the role of DCs in glioma from 1992 to 2024, thereby offering fresh perspectives for the advancement of glioma immunotherapy.

## 2 Materials and methods

### 2.1 Data sources and search strategy

**Figure 1:**
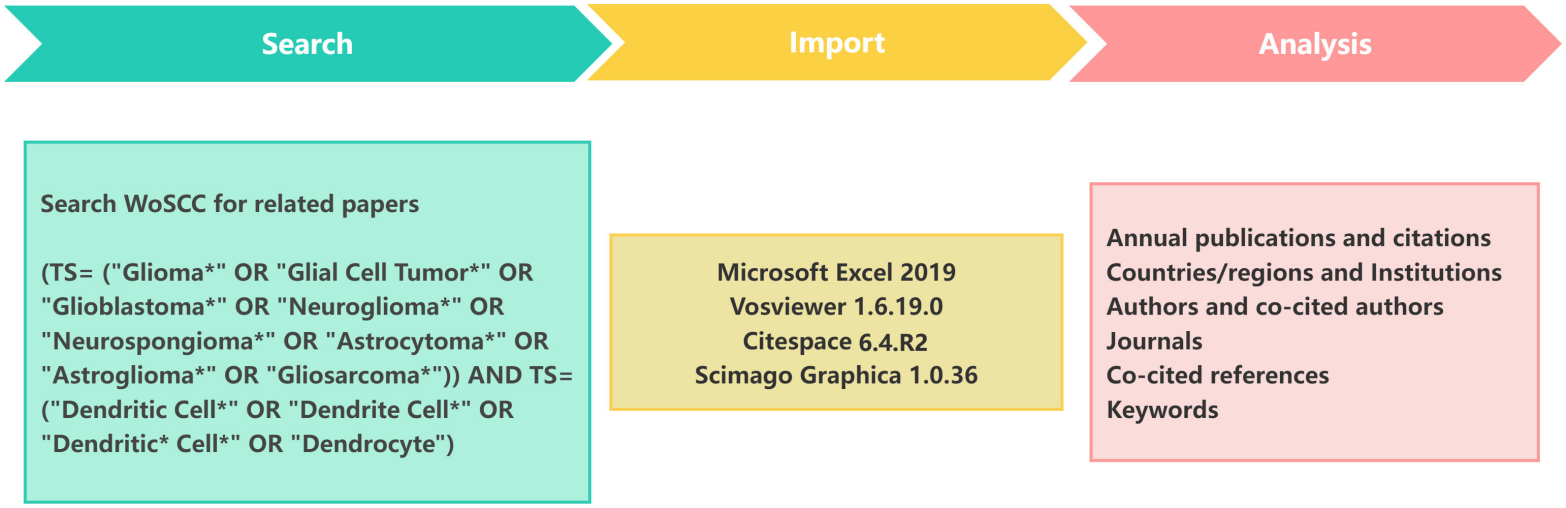
Flowchart of the literature screening and data analysis process.

The literature employed in this study was obtained from the Web of Science Core Collection (WoSCC) on 17 February 2025. Sources included the Science Citation Index-Expanded (SCI-E), the Social Sciences Citation Index (SSCI) and the Emerging Sources Citation Index (ESCI). We employed the following search strategies: (TS= (“Glioma*” OR “Glial Cell Tumor*” OR “Glioblastoma*” OR “Neuroglioma*” OR “Neurospongioma*” OR “Astrocytoma*” OR “Astroglioma*” OR “Gliosarcoma*”)) AND TS= (“Dendritic Cell*” OR “Dendrite Cell*” OR “Dendritic* Cell*” OR “Dendrocyte”). This approach yielded a total of 1875 records. We refined our selection to include only articles and review papers written in English, excluding records from 2025 to streamline the data processing. The final dataset included 1576 records spanning 1992 to 2024. Of these, articles (1,107) made up 70.24% of the total, while reviews (469) accounted for 29.76% (**Figure 1**).

### 2.2 Data analysis

For the data analysis, we employed Microsoft Excel 2019, VOSviewer 1.6.19.0 and CiteSpace 6.4.R2 to conduct a visual analysis. A variety of metrics were examined, including the number of annual publications, the H-indexes of countries and authors and the number of co-citations. VOSviewer and CiteSpace were employed to create visual representations of the distribution of countries and regions, authors, co-cited authors, institutions, journals, co-cited journals, references and keywords. Subsequently, the keywords and co-cited references were analyzed for clustering and burst detection. The clustering labels were generated using the three algorithms available in Citespace software, namely log-likelihood ratio (LLR), latent semantic indexing (LSI) and mutual information(MI) (12).

## 3 Results

### 3.1 Annual quantitative distribution of publications

**Figure 2:**
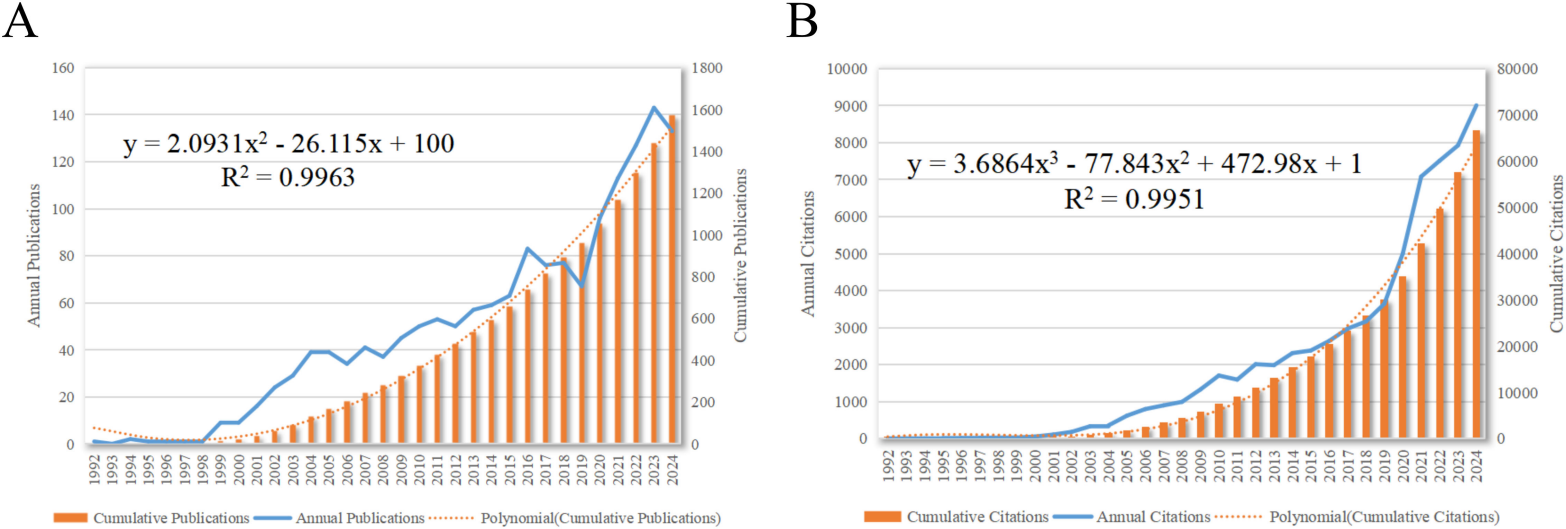
Articles related to the study of dendritic cells in glioma. **(A)** The annual and cumulative publication numbers from 1992 to 2024. **(B)** Annual and cumulative number of citations.

A total of 1,576 articles were included in this analysis. The total number of non-self-cited citations across these articles was 57,232, with an average of 42.84 citations per article. The H-index for all articles was 118. **Figures 2A, B** delineated the annual publication count and annual citation count for articles related to dendritic cells in glioma.

As depicted in **Figure 2**, the inaugural research article in this field was published in 1992. The period from 1992 to 1998 marked the beginning of research on DCs in glioma, with a relatively low volume of publications. The development phase from 1998 to 2016 was characterized by a steady increase in annual publications. The explosion phase from 2016 to 2024 witnessed a substantial surge in publications on dendritic cells in glioma, accounting for 58.06% of the total publications. This trend reflects the escalating interest among researchers in the potential role of dendritic cells in glioma research.

### 3.2 Country and regional distribution

**Figure 3:**
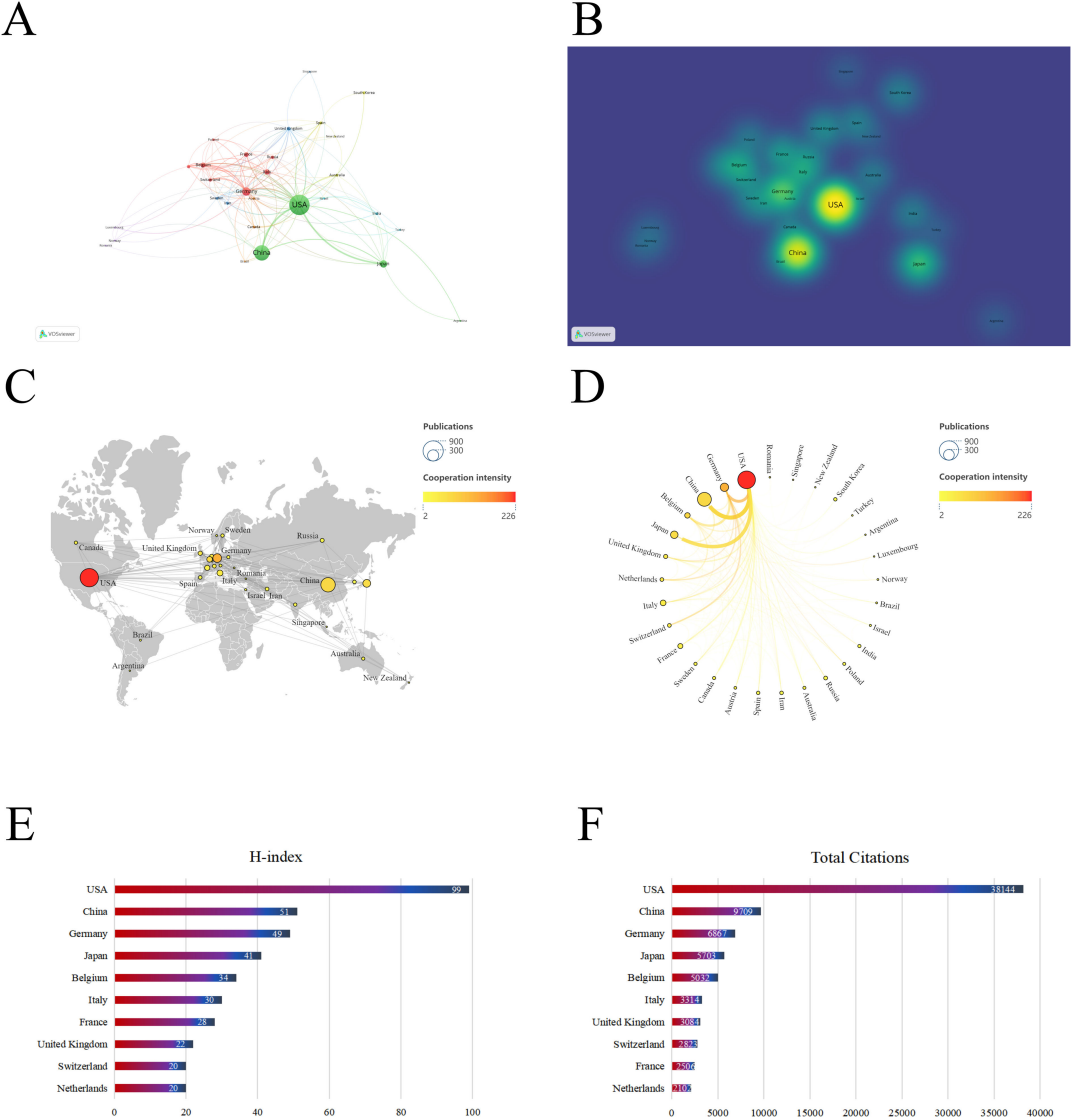
Contribution of different countries to the study of dendritic cells in glioma. **(A)** The network of collaboration map of countries/regions based on VOSviewer. **(B)** The density visualization of all participating countries. **(C)** Geographical distribution map of global publications related to dendritic cells in glioma. **(D)** The top 30 countries with the most publications. **(E)** Top 10 countries for H index. **(F)** Top 10 countries in terms of co-citation frequency.

Between 1992 and 2024, research articles on dendritic cells in glioma were published from 59 different countries/regions, as shown in **Figures 3A, B**, and detailed in **Table 1**. The United States (USA) had the largest number of publications (667/42.32%), followed by China (410/26.02%) and Germany (148/9.4%). The USA also led in citation count, amassing 38,144 citations, with China (9,709) and Germany (6,867) trailing behind. **Figures 3C, D** highlight the USA and China as dominant contributors. The node colors and line thickness in the figures denote the intensity of collaboration between countries, revealing strong collaborations between the USA and China, the USA and Japan, and the USA and Germany. The H-index, a novel method for assessing academic contributions, was also statistically analyzed. As shown in **Figures 3E, F**, the USA had the highest H-index (99), followed by China (51) and Germany (49). Taken together, these indices suggest that the USA holds a leading position in this field of research.

**Table 1:** Top 10 countries with the most published research on dendritic cells in glioma.

Rank	Country	Publications	Total citations	Average citation	H-index	Total link strength
1	USA	667	38144	57.19	99	226
2	China	410	9709	23.68	51	72
3	Germany	148	6867	46.40	49	132
4	Japan	119	5703	47.92	41	60
5	Italy	70	3314	47.34	30	36
6	Belgium	63	5032	79.87	34	64
7	France	54	2506	46.41	28	30
8	United Kingdom	37	3084	83.35	22	45
9	Russia	32	951	29.72	11	15
10	Netherlands	30	2102	70.07	20	37
10	Switzerland	30	2823	94.10	20	34

### 3.3 Authors and co-cited authors

#### 3.3.1 Authors

**Figure 4:**
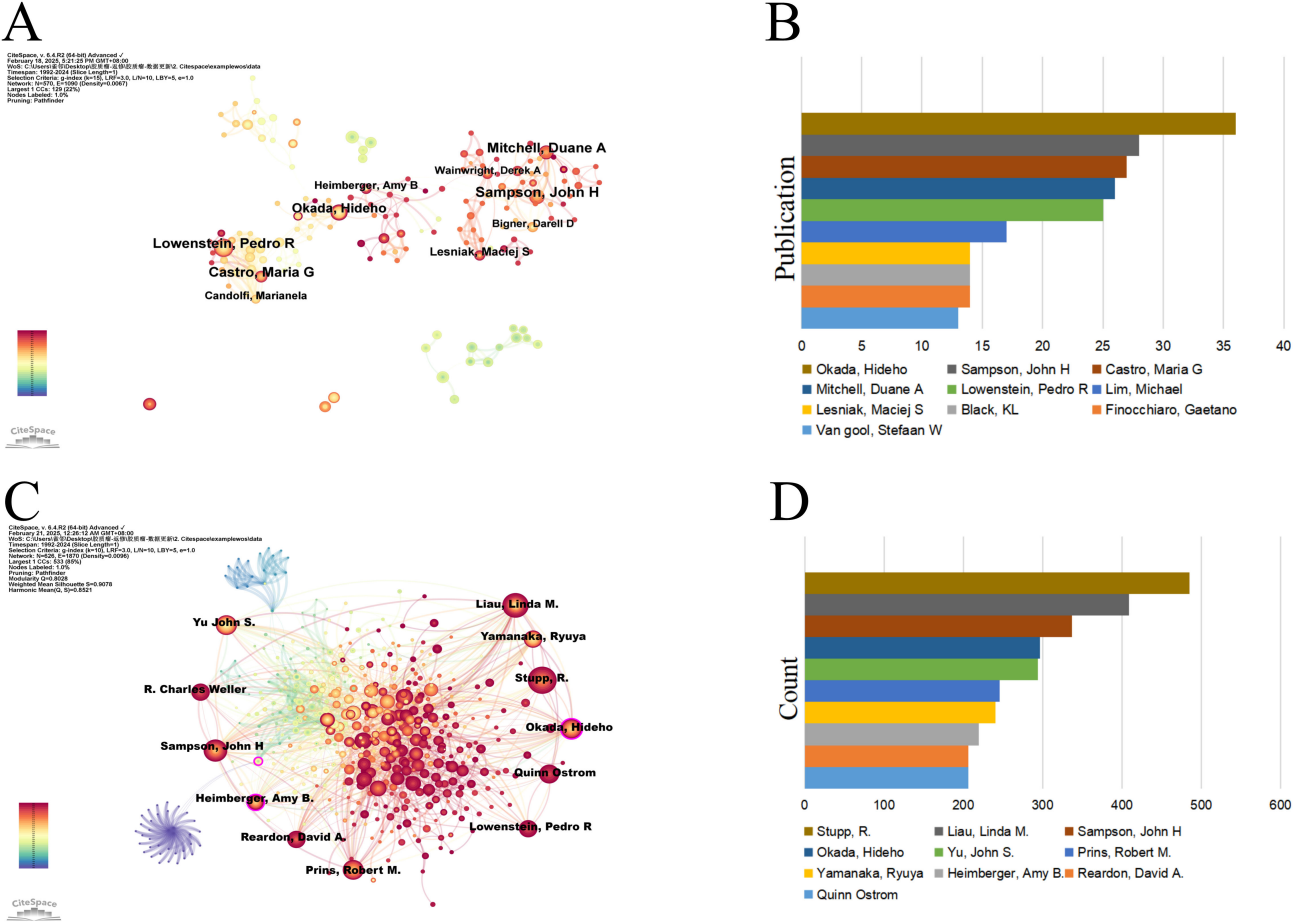
Authors involved in the study of dendritic cells in glioma. **(A)** Visualization of the co-occurrence of authors based on CiteSpace. **(B)** Top 10 authors in terms of the number of publications. **(C)** A visual map of co-cited authors based on CiteSpace. **(D)** Top 10 authors in terms of co-citations.

A visual analysis of the authors publishing articles about dendritic cells in glioma was conducted using CiteSpace, where each node symbolises an author and the size of the node correlates with the number of publications. The resulting map (**Figure 4A**) included 570 nodes and 1,090 edges, yielding a network density of 0.0067. As depicted in **Figure 4B** and **Table 2**, Okada, Hideho emerged as the most prolific author with 36 publications, closely followed by Sampson, John H (28 publications), Castro, Maria G (27 publications), and Mitchell, Duane A (26 publications). The interconnections between nodes signify collaborative relationships between authors, with line thickness indicating the degree of collaboration. The most collaborative teams were led by Okada, Hideho from the University of Pittsburgh and Sampson, John H from Duke University. However, most authors appear as isolated nodes, indicating a need for increased collaboration within this research community.

**Table 2:** Top 10 authors with the most published research on dendritic cells in glioma.

Rank	Author	Institutions	Publications
1	Okada, Hideho	University of Pittsburg	36
2	Sampson, John H	Duke University	28
3	Castro, Maria G	University of California Los Angeles	27
4	Mitchell, Duane A	University of Florida	26
5	Lowenstein, Pedro R	University of Michigan	25
6	Lim, Michael	Johns Hopkins University	17
7	Finocchiaro, Gaetano	Fondazione IRCCS Istituto Neurologico Carlo Besta	14
8	Black, KL	Cedars Sinai Medical Center	14
9	Lesniak, Maciej S	Northwestern University	14
10	Van gool, Stefaan W	Catholic University of Leuven	13

#### 3.3.2 Co-cited authors

An author is defined as co-cited author when two or more authors are cited together in at least one article, suggesting a convergence in their research themes. **Figure 4C** delineates an assemblage of all co-cited researchers, recognized as a co-citation network using CiteSpace. The ten most frequently co-cited authors each received over 200 citations (**Figure 4D**, **Table 3**). Stupp, R. was the most co-cited author with 485 citations, followed by Liau, Linda M. (409) and Sampson, John H (337). The highest centrality was attained by Paul Kleihues (0.15), Okada, Hideho (0.12), Sampson, John H (0.10), and Patrick Y. Wen (0.10) suggesting these authors serve as pivotal connectors within the field.

**Table 3:** Top 10 co-cited authors on dendritic cells in glioma.

Rank	Author	Institutions	Count
1	Stupp, R.	Northwestern University	485
2	Liau, Linda M.	University of California Los Angeles Medical Center	409
3	Sampson, John H	Duke University	337
4	Okada, Hideho	University of Pittsburg	297
5	Yu, John S.	Cedars Sinai Medical Center	294
6	Prins, Robert M.	David Geffen School of Medicine at UCLA	246
7	Yamanaka, Ryuya	Kyoto Prefectural University of Medicine	241
8	Heimberger, Amy B.	Northwestern University	220
9	Quinn Ostrom	Duke University	206
10	Reardon, David A.	Dana-Farber Cancer Institute	206

### 3.4 Active institutions

**Figure 5:**
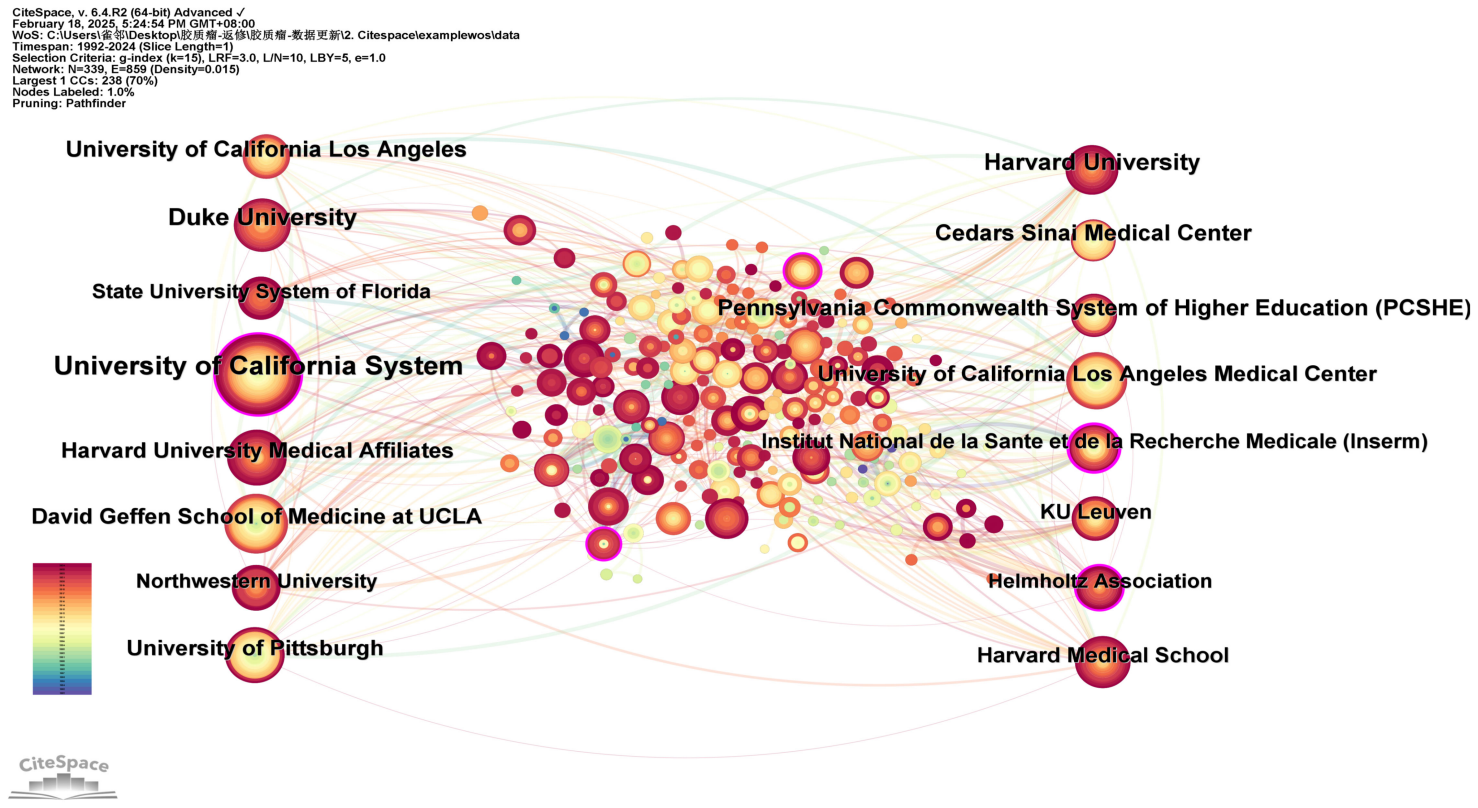
The network of institutions conducting research related to dendritic cells in glioma.

A network analysis of institutional co-occurrence was conducted using CiteSpace (**Figure 5**) to identify organizations or institutions at the forefront of dendritic cell research in glioma. The top ten institutions, ranked by the number of publications and their centrality, are listed in **Table 4**. The nodes in the figure symbolize institutions, with larger nodes denoting a greater number of publications from that institution. The links between nodes illustrate inter-institutional cooperation, with the colour of the links denoting the initiation of partnerships, and the thickness of the lines representing the intensity of the collaboration. The University of California System leads with 108 publications, followed by Duke University (62 publications) and Harvard University (56 publications). A purple circle around a node signifies a high degree of centrality (≥0.10), suggesting the institution is well-positioned to make transformative discoveries and serve as a bridge in the network. The institutions with the highest centrality are Institut National de la Sante et de la Recherche Medicale (Inserm) (0.23), NIH National Cancer Institute (NCI) (0.14), University of California System (0.13), the Helmholtz Association (0.13), and University of Texas System (0.11). The figure illustrates strong cooperation between these institutions.

**Table 4:** The top 10 institutions conduct dendritic cells in glioma by volume and centrality.

Rank	Institutions	Count	Rank	Institutions	Centrality
1	University of California System	108	1	Institut National de la Sante et de la Recherche Medicale (Inserm)	0.23
2	Duke University	62	2	NIH National Cancer Institute (NCI)	0.14
3	Harvard University	56	3	University of California System	0.13
4	University of California Los Angeles	51	4	Helmholtz Association	0.13
5	Pennsylvania Commonwealth System of Higher Education (PCSHE)	50	5	University of Texas System	0.11
6	Cedars Sinai Medical Center	50	6	Duke University	0.08
7	David Geffen School of Medicine at UCLA	47	7	Chinese Academy of Sciences	0.07
8	University of California Los Angeles Medical Center	45	8	Pennsylvania Commonwealth System of Higher Education (PCSHE)	0.06
9	University of Pittsburgh	44	9	KU Leuven	0.06
10	Harvard University Medical Affiliates	44	10	German Cancer Research Center (DKFZ)	0.06

### 3.5 Journals

**Figure 6:**
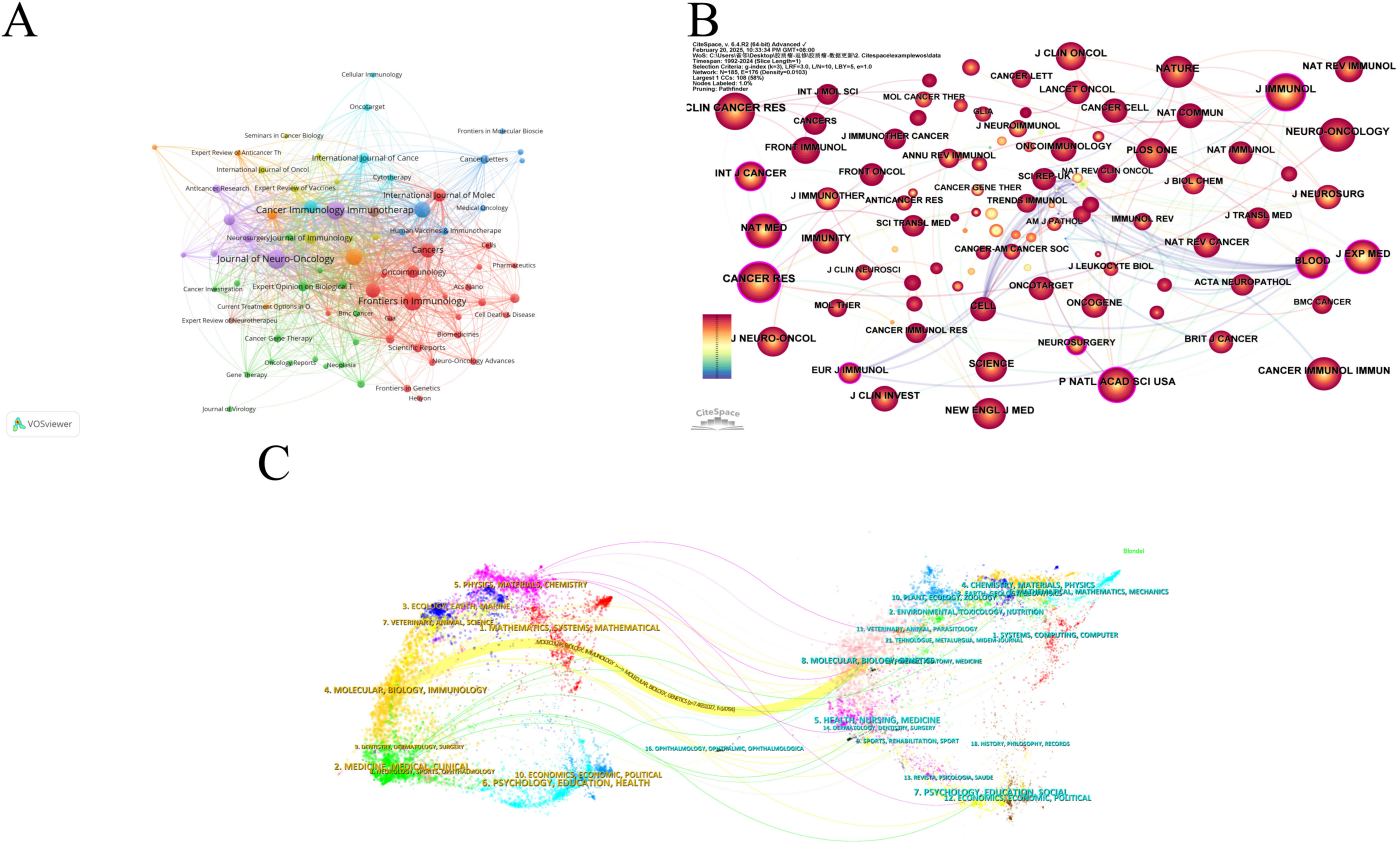
Visualization of journals of dendritic cells in glioma. **(A)** Network visualization analysis of source journals based on VOSviewer. **(B)** Visualization of cited journals based on CiteSpace. **(C)** The dual map overlay of journals in dendritic cells in glioma.

The analysis of the source literature revealed that the journal with the highest number of publications in this field was Journal of Neuro-Oncology with 54 publications. These were closely followed by Frontiers in Immunology and Cancer Immunology Immunotherapy, with 53 and 51 publications, respectively (**Figure 6A**). The Journal Citation Reports (JCR) rankings of the top 10 journals with the highest publication counts were almost in the first (Q1) or second quartile (Q2), demonstrating the reliability of the publications included in this study. This suggests that these journals could be prioritized by researchers for future submissions.

**Figure 6B** presents the co-citation analysis of journals, with Cancer Research leading the list at 1,227 citations. Clinical Cancer Research and Journal of Immunotherapy followed with 1,111 and 1026 citations, respectively. Among the top 10 most-cited journals, Nature Medicine had the highest Impact Factor (IF) of 58.7 (2024). Notably, 80% of these top-cited journals were classified as Q1, with the remaining 20% falling into the Q2 category (**Table 5**). Cancer Research emerged as the journal with the highest centrality (0.23), followed by Journal of Experimental Medicine (0.20) and Neurosurgery (0.19), highlighting the influential role these journals play in the field (**Table 5**).

**Table 5:** Top 10 journals for co-citation of dendritic cells in glioma.

Rank	Journal	Count	JCR Partitions	Rank	Journal	Centrality	JCR Partitions
1	Cancer Research	1227	Q1	1	Cancer Research	0.23	Q1
2	Clinical Cancer Research	1111	Q1	2	Journal of Experimental Medicine	0.20	Q1
3	Journal of Immunology	1026	Q2	3	Neurosurgery	0.19	Q1
4	Proceedings of the National Academy of Sciences of the United States of America	958	Q1	4	International Journal of Cancer	0.16	Q1
5	Nature	909	Q1	5	Blood	0.16	Q1
6	Neuro-Oncology	856	Q1	6	Proceedings of the National Academy of Sciences of the United States of America	0.12	Q1
7	Nature Medicine	842	Q1	7	Nature Medicine	0.12	Q1
8	Journal of Experimental Medicine	820	Q1	8	European Journal of Immunology	0.11	Q2
9	Cancer Immunology Immunotherapy	817	Q1	9	Journal of Immunology	0.10	Q2
10	Journal of Neuro-Oncology	777	Q2	10	Journal of Immunotherapy	0.10	Q2

The dual map overlay in CiteSpace provides insights into the evolution of research across various disciplines (**Figure 6C**). The left side displays citing articles, while the right side shows cited articles. The coloured, curved path in the centre represents citation relationships. The yellow citation path indicates that research published in journals within the Molecular/Biology/Genetics categories are frequently cited by Molecular/Biology/Immunology journals. Furthermore, peripheral disciplines such as Physics/Materials/Chemistry, Ecology/Earth/Marine, Veterinary/Animal/Science, Environmental/Toxicology/Nutrition, and Psychology/Education/Social are also engaged in dendritic cell research in glioma. This underscores the multidisciplinary and collaborative nature of research in this field.

### 3.6 Co-cited references

**Figure 7:**
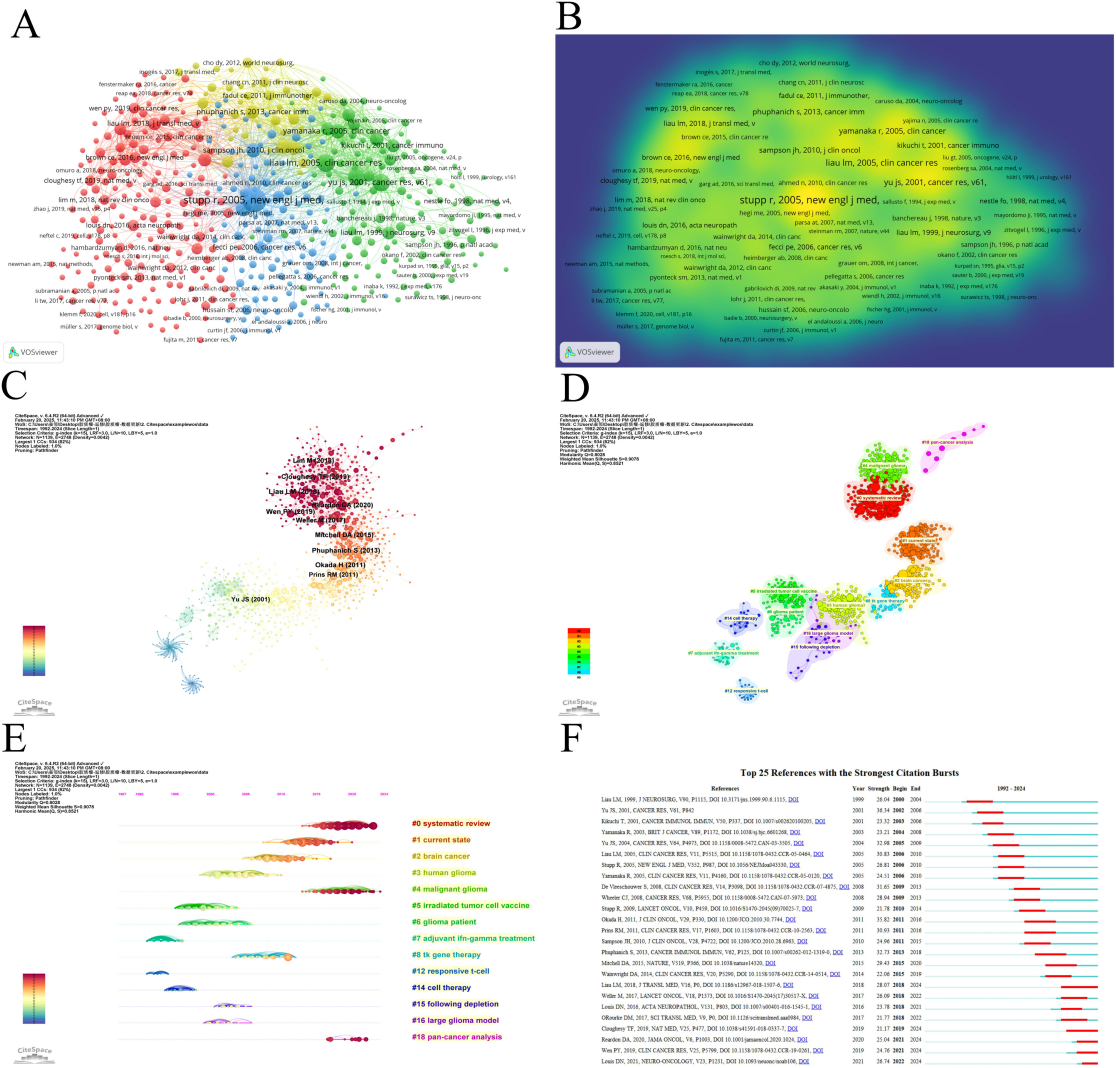
Visualization of co-cited literature on dendritic cells in glioma. **(A)** References co-citation network in dendritic cells in glioma based on VOSviewer. **(B)** The density visualization of co-cited references based on VOSviewer. **(C)** References co-citation network in dendritic cells in glioma based on CiteSpace. **(D)** Cluster Analysis of Co-cited References based on CiteSpace. **(E)** Top 25 references with the strongest citation bursts in dendritic cells in glioma. **(F)** A timeline of the 15 largest clusters in dendritic cells in glioma.

Co-cited references illustrate the level of interconnection between different sources. Utilizing VOSviewer, we identified the top five references with the highest co-citation frequencies (**Figures 7A, B**, **Table 6**). Notably, the most frequently co-cited article was authored by Stupp, R., which demonstrated the significant survival benefit of introducing temozolomide early in radiotherapy for glioblastoma treatment (1). However, the quest to further enhance clinical outcomes remains an ongoing challenge (13). To further analyze the co-cited references, we employed CiteSpace with the following parameters: time slicing (1992–2024), years per slice (1), node type (cited reference), selection criteria (k=15), and pruning (Pathfinder, Pruning sliced networks&Pruning the merged network). As depicted in **Figure 7C**, this analysis generated a co-occurrence network comprising 1,139 nodes, 2,748 connections, and a density of 0.0042. Subsequently, we conducted a cluster analysis on the cited references using the LSI, yielding 97 clusters. The top 14 clusters are displayed in **Figures 7D, E**. Among these, the clustering modularity Q was 0.8028, and the average silhouette score S was 0.9078, suggesting a well-structured clustering with a significant cohesion. The value of the cluster number signifies the level of interest in the cluster topic within the discipline, with a smaller cluster value indicating higher attention. These clusters can be categorized into 4 main areas (**Figure 7E**). Firstly, research background and context, including (#1 current state, #2 brain cancer, #3 human glioma, #4 malignant glioma). Secondly, therapeutic strategies and approaches, including (#5 irradiated tumor cell vaccine, #7 adjuvant ifn-gamma treatment, #8 tk gene therapy, #14 cell therapy). Followed by immune responses and mechanisms, including (#12 responsive T-cell, #15 following depletion, #16 large glioma model), and finally, analysis and synthesis, including (#0 systematic review, #18 pan-cancer analysis).

**Table 6:** The top 5 most co-cited references related to dendritic cells in glioma.

First Author	Journal	Year	Citations	TLS
Stupp, R.	New England Journal of Medicine	2005	376	7792
Liau, Linda M.	Clinical Cancer Research	2005	218	5877
Yu, John S.	Cancer Research	2001	206	4844
Yu, John S.	Cancer Research	2004	201	4969
Stupp, R.	Lancet Oncology	2009	151	4088

TLS, Total link strength.

Burst detection of cited references signifies a shift in research focus within a field. In CiteSpace, we set the parameters minimum Duration=2, g=1, and identified 25 references with significant citation bursts, underscoring their relevance in the field of dendritic cells in glioma (**Figure 7F**). Among these, seven papers published between 2017 and 2021 primarily focus on GBM, except for the 2021 article titled “The 2021 WHO classification of tumours of the central nervous system a summary” This article provides an updated overview of the classification system for CNS tumours, including GBM (14). The remaining articles concentrate on GBM treatment, predominantly through randomized, double-blind, controlled trials. They report on the results of clinical trials and explore immunotherapy in the treatment of GBM. For example, O’Rourke DM et al.(2017) shared initial experiences with chimeric antigen receptor (CAR) T cells in recurrent GBM, suggesting that although intravenous infusion exhibits on-target activity in the brain, enhancing the efficacy of EGFRvIII-directed strategies may require addressing the antigen heterogeneity and adaptive changes in the local tumour microenvironment (15). In another 2017 study, Michael Weller’s team conducted a Phase III clinical trial examining a vaccine called Rindopepimut (also known as CDX-110) that targets EGFRvIII, a mutated form of the epidermal growth factor receptor (EGFR). The findings indicate that diverse approaches could enhance the effectiveness of immunotherapy for GBM (16). A 2018 article corrects the initial survival results of a Phase III clinical trial using an autologous DC vaccine in newly diagnosed GBM patients, indicating that the addition of DCVax-L to standard therapy is feasible and safe in glioblastoma patients and may extend survival (17). In a 2019 article, Wen PY et al. reported that progression-free survival (PFS) significantly improved in patients treated with ICT-107, maintaining quality of life (QoL), particularly in the HLA-A2 subgroup, which showed elevated ICT-107 activity both in clinical and immunological outcomes (18). Another 2019 study explored neoadjuvant anti-PD-1 immunotherapy for recurrent GBM, suggesting that neoadjuvant therapy with PD-1 blockade enhances local and systemic anti-tumour immune responses, potentially offering a more effective treatment for this aggressive brain tumour (19). The 2020 article presents the findings from the CheckMate 143 phase III trial, comparing nivolumab and bevacizumab in patients with recurrent GBM. Although the primary endpoint was not reached, median overall survival (mOS) was similar between the nivolumab and bevacizumab treatment groups in patients with recurrent glioblastoma. The safety profile of nivolumab in glioblastoma patients was comparable to that previously observed in patients with other tumours (20).

### 3.7 Keyword analysis

**Figure 8:**
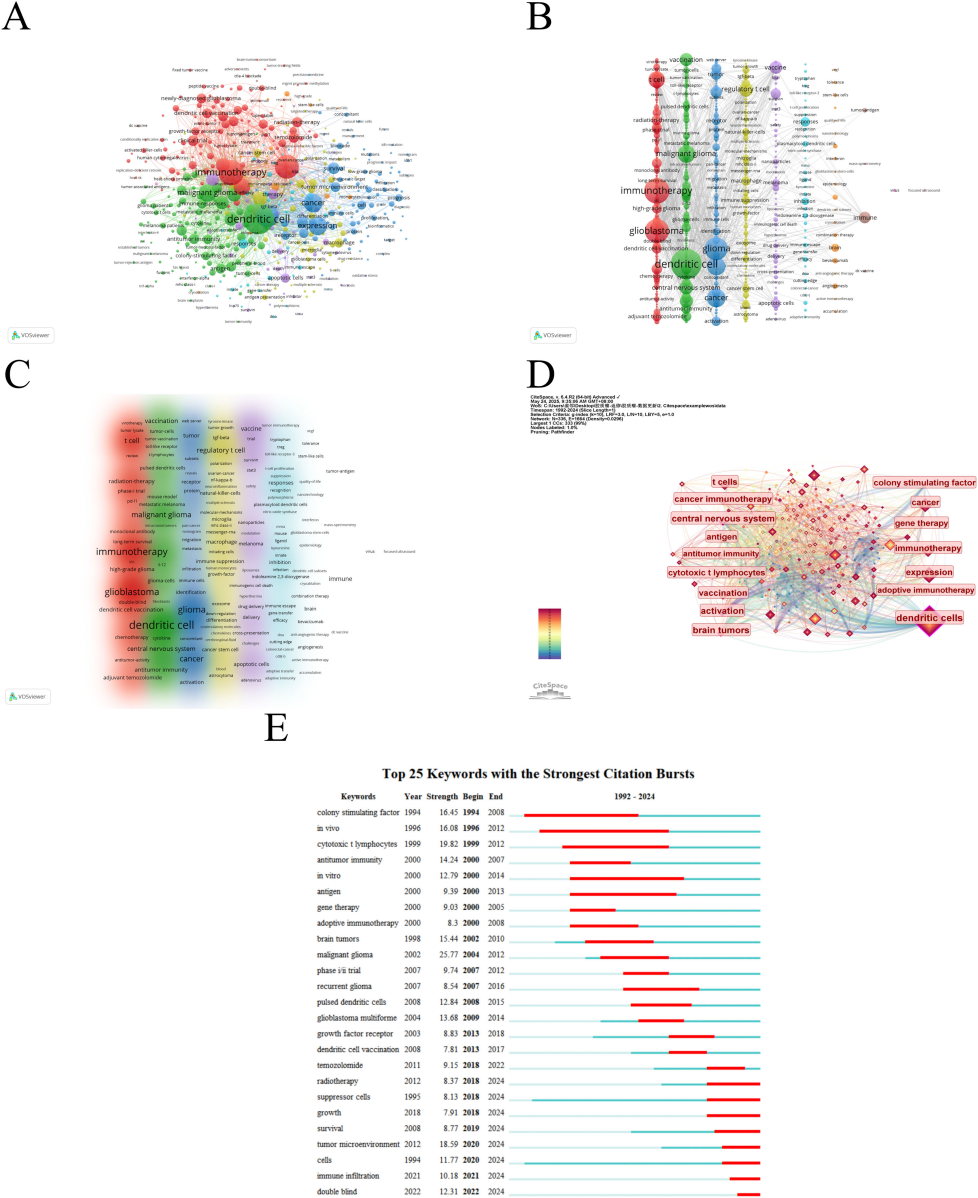
The mapping on keywords of dendritic cells in glioma. **(A)** Network map of 490 keywords with frequency more than 5. **(B)** The cluster of keywords in the studies of dendritic cells in glioma (divided into 9 clusters by different colors. **(C)** Density visualization of keyword clustering. **(D)** Visualization of keywords based on CiteSpace. **(E)** Top 25 keywords with the strongest citation bursts.

A keyword co-occurrence network can facilitate the identification of research hotspots and trends within a specific field. In this study, we utilized VOSviewer software for keyword analysis, setting the minimum occurrence of a keyword to five, which yielded a total of 490 keywords (**Figure 8A**, **Table 7**). Subsequent cluster analysis revealed nine distinct clusters (**Figures 8B, C**), encompassing seven research directions and areas of study. The largest cluster is the cluster 1 (red), with 105 keywords, including immunotherapy, glioblastoma, t cell, glioblastoma multiforme, temozolomide, clinical trial, radiation-therapy, dendritic cell vaccination, newly diagnosed glioblastoma, phase-ii trial, etc. Cluster 2 (green) includes 102 keywords, such as dendritic cell, malignant glioma, central nervous system, brain tumor, vaccination, antitumor immunity, cytotoxic t lymphocyte, antigen, *in-vitro*, cancer immunotherapy, etc. Cluster 3 (blue) also has 92 keywords, mainly including glioma, cancer, expression, survival, tumor, tumor microenvironment, activation, cell, receptor, identification, etc. Cluster 4 (yellow) contains 82 keywords, focusing on regulatory t cell, macrophage, blood brain barrier, immune suppression, suppressor-cells, cancer stem cell, natural-killer-cells, tumor-associated macrophages, microglia, differentiation. Cluster 5 (purple) has 51 keywords, mainly related to vaccine, therapy, apoptotic cells, melanoma, delivery, mechanism, nanoparticles, trial, drug delivery, cross-presentation. Cluster 6 (light blue), with 37 keywords, primarily features responses, inhibition, plasmacytoid dendritic cells, efficacy, indoleamine 2,3-dioxygenase, immune escape, ligand, cutting edge, aryl-hydrocarbon receptor, innate. Cluster 1 and 5 mainly reflect glioblastoma immunotherapy and clinical trials, while Cluster 2 and 6 mainly emphasize dendritic cells and antitumor immunity. Cluster 3 and 4 mainly reflect tumor microenvironment and immune regulation. In addition, we utilized CiteSpace to visualize the keyword co-occurrence network, generating a map with 336 nodes, 1664 connections and a density of 0.0296 (**Figure 8D**). Burst detection analysis identified that recent studies on ‘double blind’, ‘immune infiltration’, ‘cell’, and ‘tumour microenvironment’ are the most current keywords (**Figure 8E**), signifying these areas as recent research hotspots and potential future research focuses.

**Table 7:** Top 10 keywords in terms of frequency in dendritic cells in glioma.

Rank	Keywords	Occurrences	TLS
1	Dendritic Cell	852	7171
2	Immunotherapy	577	5360
3	Glioblastoma	520	4579
4	Glioma	469	4245
5	Cancer	323	2671
6	Expression	300	2438
7	Malignant Glioma	279	2547
8	T Cell	245	2264
9	Regulatory T Cell	179	1710
10	Glioblastoma Multiforme	175	1628

TLS, Total link strength.

## 4 Discussion

### 4.1 General information

This investigation analyzed 1,576 articles on dendritic cells in glioma retrieved from the Web of Science core database, using VOSviewer, CiteSpace and Excel. Over the past 33 years, the publication of papers in this field has generally trended upwards, with the annual average co-citations similarly increasing. Prior to 1998, this area of study was relatively unexplored. However, after 1998, research on the role of dendritic cells in glioma gained increasing attention, particularly from 2016 onwards, when the field began to reach a stage of relative maturity. A total of 1,576 articles were included, revealing a significant annual increase in dendritic cell-focused glioma research. The USA, China, and Germany led in publication output. Okada, Hideho had the most publications, while Stupp, R had the highest co-citations. Journal of Neuro-Oncology published the most articles, and Cancer Research received the highest citations. The analysis highlights key themes such as “dendritic cell”, “immunotherapy”, and “glioblastoma”, and emerging interests like “tumor microenvironment”, “immune infiltration”, and “double blind”. Details are as follows:

### 4.2 Key contributing countries and prominent authors

Currently, the USA, China and Germany are the leading nations conducting research on dendritic cells in glioma (**Figure 3**). Among them, the USA stands out with the highest number of publications, citations, H-index and TLS with an average citation rate of 57.19, indicating its dominance in both quantity and quality. Despite China ranks second in terms of published articles, its average citation of 23.68 suggests there is room for improvement in producing high-quality papers. Furthermore, five of the top 10 institutions by centrality are based in the USA, underscoring its pivotal role in the global collaboration network (**Table 4**). **Figure 3C, D** reveal particularly strong cooperative relationships between the USA and China, the USA and Japan, as well as the USA and Germany. The strength of connections between nodes indicates that enhanced international cooperation could further accelerate progress in this area.

In terms of authorship, Okada, Hideho leads in both publications and contributions to dendritic cell-based immunotherapy for glioma. His phase I/II clinical trial on a poly-ICLC-boosted αDC1-based vaccine for patients with recurrent malignant glioma showed promising safety, immunogenicity, and preliminary clinical activity. Notably, Okada, Hideho is among the top 10 co-cited authors, with a centrality value exceeding 0.1, further demonstrating his significant position in the research field of dendritic cells and glioma (21). R. Stupp, the most frequently co-cited researcher, is an authority in Neuro-Oncology. His research interests encompass primary and secondary brain tumors, head and neck cancers, drug discovery, chemotherapy and radiation therapy. Stupp and his team have shown that Tumor Treating Fields improve survival rates in patients with glioblastoma (22). John H. Sampson also boasts a strong publication and co-citation record, particularly for his team’s development of Cytomegalovirus (CMV) pp65 DC vaccines, which have shown consistent efficacy in three consecutive clinical trials for glioblastoma (23).

### 4.3 Hotspots and frontiers

#### 4.3.1 “Immune infiltration” and “cells” as emerging research frontiers in glioma

Burst detection analysis of keywords provides a visual representation of research frontiers (24), with “immune infiltration” emerging as a focal point in the last four years. Immune infiltration refers to the process by which immune cells penetrate tumor tissue (25), encompassing a variety of immune cells, including T cells, B cells, macrophages and dendritic cells. These cells play a crucial role in both tumor progression and regression. It forms the foundation of immunotherapies such as immune checkpoint inhibitors and CAR-T cell therapy, which aim to augment or re-activate the patient’s immune response to attack tumor cells (26). In gliomas, the extent of immune cell infiltration can serve as a prognostic indicator (27), with higher levels of infiltration generally correlating with better outcomes. Therefore, comprehensive studies of immune infiltration may facilitate the identification of novel therapeutic targets, thereby promoting the development of more effective treatment strategies (28).

Additionally, “cells” also emerged as a major research frontier in burst detection analysis, reflecting growing interest in various cellular components such as neuron, dendritic cells, astrocytes and microglia involved in glioma biology. A team from Zhejiang University School of Medicine established a direct correlation between olfactory sensory neuron activity in the olfactory neural circuit and glioma development, revealing that olfactory stimulation can directly modulate malignant glioma development via activation of the IGF1 signaling pathway (29). Furthermore, astrocytes, which are unique to the CNS and can form functional gap junctions with glioma cells, facilitating the transfer of signaling molecules and supporting glioma growth (30). Moreover, microglia, the resident immune cells of the CNS, also play a significant role in glioma biology. They can be activated by glioma cells and release pro-inflammatory and pro-tumorigenic factors, further contributing to glioma progression (31). Notably, the crosstalk between dendritic cells and glioma cells has been shown to play a crucial role in glioma progression. Dendritic cells, as potent antigen-presenting cells, can present tumor antigens to T cells to elicit an anti-tumor immune response. However, in the glioma microenvironment, tumor cells can release immunosuppressive factors that impair the function of dendritic cells, leading to immune evasion (32).

#### 4.3.2 The role of dendritic cells in the tumor microenvironment

The application of cluster analysis to keywords and co-cited references provides researchers with a comprehensive understanding of current research trends in a given field (33). Among these trends, dendritic cells, as the most efficient and specialized antigen-presenting cells, have emerged as critical players in tumor immunotherapy (**Table 7**), especially in the context of the tumor microenvironment (**Figure 8E**). Under physiological conditions, DCs activate helper T cells, cytotoxic T cells, and NKcells by capturing and presenting tumor antigens, expressing co-stimulatory molecules (34), and migrating to lymphoid organs to initiate immune responses (35). However, within the immunosuppressive glioma microenvironment, DCs often remain in an immature or dysfunctional state, leading to impaired antigen presentation and the induction of immune tolerance rather than activation. This dual role—promoting either anti-tumor immunity or immune suppression depending on the tumor microenvironment—has sparked considerable interest in understanding and manipulating DC function for therapeutic purposes (36).

In the glioma microenvironment, DCs are rendered dysfunctional by various factors. Immunosuppressive cytokines such as transforming growth factor-beta (TGF-β) and IL-10 inhibit their maturation (37), while glioma-derived exosomes disrupt their differentiation (38). Hypoxia-induced HIF-1α in DCs can upregulate IDO and PD-L1, suppressing T cell activation and promoting exhaustion (39). Additionally, regulatory T cells (Tregs) and myeloid-derived suppressor cells (MDSCs) directly or indirectly suppress DC functions through inhibitory cytokines (40).

#### 4.3.3 Therapeutic potential of dendritic cell vaccines in gliomas

Gliomas, prevalent malignant tumors of the central nervous system, present considerable therapeutic challenges, including poor prognosis, high recurrence rates and mortality (41). Currently, clinical interventions are limited, with median survival for patients only exceeding 15 months. Given these challenges, increasing evidence suggests that dendritic cell vaccines have demonstrated considerable promise as an immunotherapeutic strategy against malignant gliomas (42–44).The details are as follows:

##### 4.3.3.1 Clinical trials of dendritic cell vaccines for glioma treatment

DC vaccines leverage the antigen-presenting capabilities of DCs to stimulate an immune response against glioma cells. The preparation of this vaccine involves the *in vitro* generation of dendritic cells. These cells were isolated from peripheral blood or differentiated from monocytes or CD34+ hematopoietic progenitor cells *in vitro*. Then, they were expanded *in vitro*, exposed to tumor-associated antigens, and reintroduced into the patient’s body (45).

Currently, DC vaccines such as DCVax-L and cytomegalovirus phosphoprotein 65 RNA (CMV pp65) are being tested in clinical trials for various cancers, exhibiting notable effects (46). DCVax-L is a kind of dendritic cell with tumor lysate antigen. Notably, a phase III clinical trial of the DCVax-L vaccine revealed a significant improvement in patient survival (47). The median overall survival escalated to 19.3 months, compared to 16.5 months in the control group (48). Moreover, the 5-year survival rate for the DCVax-L group was 13.0%, a marked improvement over the 5.7% observed in the control group (49). Another noteworthy clinical trial involved the CMV pp65 dendritic cell vaccine. This discovery led to targeting the CMV antigen pp65 (50). In a phase 1 trial, 11 newly diagnosed GBM patients received the intradermal CMV pp65 dendritic cell vaccine and temozolomide. The median PFS was 25.3 months, and the median OS was 41.1 months. Both are longer than the predicted rate (51).

##### 4.3.3.2 Strategies to enhance the efficacy of dendritic cell vaccines in glioma immunotherapy

Although DC vaccines are promising for glioma immunotherapy, their efficacy remains limited due to the immunosuppressive glioma microenvironment, which impairs DC function (43, 52), and insufficient immunogenicity resulting from suboptimal antigen selection, incomplete DC maturation, and patient variability (43, 52, 53). To enhance the therapeutic potential of DCs, several strategies have been explored. One approach involves combining DC vaccines with other immunotherapies, such as immune checkpoint inhibitors (ICIs), peptide vaccines, or oncolytic virus therapy, can enhance efficacy by overcoming immunosuppression (53, 54). This combination can help overcome the immunosuppressive effects of the tumor microenvironment by blocking inhibitory pathways, thereby enhancing the efficacy of DC vaccines. For example, the use of anti-PD-1 or anti-CTLA-4 antibodies in conjunction with DC vaccines has shown synergistic effects in preclinical models (17, 18).

Another strategy focuses on improving the function of DCs within the glioma microenvironment. This can be achieved by targeting specific pathways that contribute to DC dysfunction. For instance, blocking the TGF-β pathway, which is known to inhibit DC maturation and function, has been shown to enhance the efficacy of DC vaccines (55). Additionally, the use of hypoxia-activated prodrugs can help mitigate the effects of hypoxia on DCs, thereby improving their function in the tumor microenvironment (43). Furthermore, the development of novel DC-based therapies, such as DC vaccines loaded with tumor-associated antigens (TAAs) or neoantigens, offers a personalized approach to glioma treatment (43). Moreover, the use of adjuvants, such as polyinosinic-polycytidylic acid (poly-IC), can enhance the immunogenicity of DC vaccines by stimulating the innate immune system (21).

## 5 Innovation and limitations

This study provides a comprehensive bibliometric analysis of dendritic cell research in glioma, offering novel insights into the evolving research landscape over 1992–2024. The use of advanced visualization tools (CiteSpace and VOSviewer) enables a detailed assessment of key research clusters and emerging hotspots, such as immune infiltration and dendritic cell vaccines. However, there are still several limitations to this study. Firstly, all collected publications were retrieved solely from WoSCC. Although it is one of the most reliable online databases (56), some publications may have been missed. Secondly, the analysis was limited to English-language publications, potentially omitting valuable research in other languages. Thirdly, as our study was based on published literature, this could have led to publication bias. Therefore, we expanded the search as much as possible to avoid missing literature to minimize publication bias. Finally, limitations of software such as CiteSpace and VOSviewer, there may be differences in keyword bursts, co-citation cluster analysis, and institutions related to glioma immunotherapy.

## 6 Conclusion

In summary, this bibliometric analysis examines the evolution of dendritic cell research in glioma from 1992 to 2024, highlighting key trends and future directions. The findings indicate that dendritic cells, immunotherapy, and glioblastoma remain central to this field, with emerging hotspots including immune infiltration and tumor microenvironment. Dendritic cell vaccines show promise in glioma trials but are hindered by the immunosuppressive tumor microenvironment. Future work should focus on enhancing dendritic cell function and developing synergistic combination therapies to improve outcomes.

## Data Availability

The original contributions presented in the study are included in the article/**Supplementary Material**. Further inquiries can be directed to the corresponding authors.
